# *In vivo* voltage-sensitive dye imaging of mouse cortical activity with mesoscopic optical tomography

**DOI:** 10.1117/1.NPh.7.4.041402

**Published:** 2020-12-02

**Authors:** Qinggong Tang, Vassiliy Tsytsarev, Feng Yan, Chen Wang, Reha S. Erzurumlu, Yu Chen

**Affiliations:** a University of Oklahoma, Stephenson School of Biomedical Engineering, Norman, Oklahoma, United States; b University of Maryland, Fischell Department of Bioengineering, College Park, Maryland, United States; c University of Maryland School of Medicine, Department of Anatomy and Neurobiology, Baltimore, Maryland, United States; d University of Massachusetts, Department of Biomedical Engineering, Amherst, Massachusetts, United States

**Keywords:** optical tomography, functional brain mapping, imaging three-dimensional neural activity, mesoscopic fluorescence tomography, voltage-sensitive dye imaging, optogenetics, channelrhodopsin, intercortical connections, whiskers, somatosensory system

## Abstract

**Significance:** Cellular layering is a hallmark of the mammalian neocortex with layer and cell type-specific connections within the cortical mantle and subcortical connections. A key challenge in studying circuit function within the neocortex is to understand the spatial and temporal patterns of information flow between different columns and layers.

**Aim:** We aimed to investigate the three-dimensional (3D) layer- and area-specific interactions in mouse cortex *in vivo*.

**Approach:** We applied a new promising neuroimaging method—fluorescence laminar optical tomography in combination with voltage-sensitive dye imaging (VSDi). VSDi is a powerful technique for interrogating membrane potential dynamics in assemblies of cortical neurons, but it is traditionally used for two-dimensional (2D) imaging. Our mesoscopic technique allows visualization of neuronal activity in a 3D manner with high temporal resolution.

**Results:** We first demonstrated the depth-resolved capability of 3D mesoscopic imaging technology in Thy1-ChR2-YFP transgenic mice. Next, we recorded the long-range functional projections between sensory cortex (S1) and motor cortex (M1) in mice, *in vivo*, following single whisker deflection.

**Conclusions:** The results show that mesoscopic imaging technique has the potential to investigate the layer-specific neural connectivity in the mouse cortex *in vivo*. Combination of mesoscopic imaging technique with optogenetic control strategy is a promising platform for determining depth-resolved interactions between cortical circuit elements.

## 1 Introduction

The mammalian neocortex plays an important role in higher brain function, including sensory perception, cognition, associative learning, and goal-directed motor control.^1^ Tangential to the cortical surface, the neocortex is organized in such a way that it is both highly specialized, with defined areas dedicated to specific functions and/or sensory modalities, and highly integrative, with each area receiving converging inputs from different thalamic nuclei, other cortical areas, and several neuromodulatory systems.^1^^,^^2^ Another form of cortical organization is clearly evident orthogonal to these tangentially distributed maps.^2^ The neocortex is classically divided along its depth into six anatomically defined layers, from superficial layer 1 to deep layer 6. Each layer contains distinct classes of cells that project cortically or subcortically, along with GABAergic interneuron types.^2^^,^^3^ These local neocortical microcircuits (six layers of interconnected excitatory and inhibitory neurons) will process and integrate all the area-specific inputs from different thalamic nuclei, other cortical areas, and several neuromodulatory systems.

In primary sensory cortices, information from the periphery primarily relayed by the thalamus mainly impact the layer 4 (L4), also known as the granular cell layer. L4 stellate cells mainly excite layers 2/3 (L2/3) pyramidal neurons, which in turn excite neurons in layer 5 (L5), and to the whole cortical column.^4^^,^^5^ The deep infragranular L5 and layer 6 (L6) are the main source of cortical outputs to subcortical structures (such as the thalamus, striatum, and brainstem).^1^ Cortical layers 1, 2, and 3 form the superficial layers, also known as the supragranular layers, which are likely the most integrative layers, gathering sensory information, and contributing an important source of projections to other cortical areas.^2^ Anatomical studies have shed light on the synaptic architecture of cortical microcircuits.^6^^,^^7^ The difficult challenge now is to relate the wiring diagram of these neuronal networks to their functional operation.

There has been rapid technological progress over the last decade in measuring and perturbing neuronal activity in the mouse neocortex *in vivo*. Based on blood oxygen level-dependent (BOLD) contrast, functional magnetic resonance imaging (fMRI) has revolutionized human cognitive neuroscience.^8^^,^^9^ Attempts to apply fMRI findings in mouse neocortex are prohibitively challenging, since the small size of the mouse neocortex (∼1-mm-thick in depth) necessitates exceptionally high signal-to-noise ratio (SNR) and spatial resolution.^10^ Electrophysiological methods that detect functional synapses, including paired recordings and glutamate uncaging-based methods, have been applied to map local circuits within mouse cortex. However, most of these electrophysiological techniques estimating the layer-specific connectivity in cortical circuits are limited to *ex vivo* brain slices.^4^^,^^11^^–^^15^ *In vivo* simultaneous recordings across the entire depth of the primary auditory cortex^16^ and visual cortex^17^ have been demonstrated to study the laminar specificity in spatiotemporal dynamics by inserting a sharp linear array microelectrodes, whereas these methods are labor-intensive, invasive (potentially damaging to the cortex) and can only record the action potentials of limited neural cells next to the extracellular electrodes.^18^

Optical methods have been proven to be useful in monitoring neural responses in the brain, as they offer advantages for studying functional organization.^19^ However, most of the current optical imaging setups involving charge coupled device (CCD) cameras can only provide two-dimensional (2D) information and cannot detect depth-resolved neuronal activation. In order to investigate the three-dimensional (3D) layer-specific interaction in mouse cortex and between different cortical areas *in vivo*, an imaging system with large field of view (FoV), depth-resolved ability, and high imaging speed is needed. Laser-scanning microscopy (LSM), such as confocal and two-photon microscopy, has revolutionized biomedical research with high resolution, whereas current implementations have limited imaging depth and FoV.^20^^–^^22^ Moreover, different layers have to be scanned sequentially to image 3D volumes in LSM, which makes it difficult to acquire the 3D functional responses in the brain with high temporal resolution.^20^^–^^22^ Utilizing a multitude of sources and detectors with an inversion algorithm to reconstruct the underlying tissue properties in 3D, diffuse optical tomography (DOT) has been applied to achieve 3D reconstruction of cerebral hemodynamics in small animals.^23^^–^^25^ High-density speckle contrast optical tomography has also been developed for *in vivo* 3D imaging of blood flow in small animals.^26^ However, these systems were designed to cover subcortical tissues in mouse brain thus suffered from poor spatial resolution especially in axial direction (>1  mm).

As a modified version of DOT, laminar optical tomography (LOT) was initially developed to image absorption contrast for hemodynamic changes in mesoscopic range.^27^^–^^30^ Soon after, it was adapted to fluorescent molecular imaging, termed either fluorescence laminar optical tomography (FLOT)^30^^–^^33^ or mesoscopic fluorescence molecular tomography.^34^^,^^35^ Similar to DOT,^36^ LOT uses an array of photon detectors or a CCD camera to collect photons emitted from locations at different distances away from the illumination position, enabling simultaneous detection of scattered light traveling through different depths in the tissue. By applying much denser spatial sampling, FLOT can achieve a resolution of ∼30 to 100  μm with millimeters imaging depth.^28^^,^^33^ Recent studies demonstrated that angled illumination or detection configuration can further improve both resolution and depth sensitivity.^33^^,^^37^

In order to study the specific function of each layer in the neocortex, development of technologies to regulate the type-specific cells is key to understand how they contribute to local network activity and overall brain function *in vivo*.^38^ This control has become feasible with the advent of optogenetics, in which single-gene encoding light-activated ion-conductance regulators or biochemical signaling proteins are introduced into targeted cells.^38^^–^^40^ This approach provides a reliable method for stimulating or suppressing neural activity in mammalian tissues,^12^^,^^41^^–^^47^ and researchers can now control activity in defined neuronal populations and projections while examining the consequences on behavior and physiology.^40^

In this paper, we demonstrate the depth-resolved capability of a mesoscopic optical imaging technique with angled illumination configuration for *in vivo* brain functional imaging in mouse cortex. Rather than optical intrinsic contrast, we applied voltage-sensitive dye (VSD), an exogenous fluorescent dye to provide specific optical contrast. VSD can report changes in membrane potential by binding to the neural membrane and converting changes in transmembrane voltage into the signal changes of the emitted fluorescence light.^48^^,^^49^ Compared to hemodynamics imaging based on absorption contrast (slow signal changes at the second time scale), voltage-sensitive dye imaging (VSDi) provides a reliable and direct measures of neural activity in the brain with relatively high spatial and temporal resolution (at the millisecond time scale).^48^^,^^50^ We first probed the neural connection in primary sensory cortex in Thy1-ChR2-YFP transgenic mice. We further imaged the layer-specific functional projections between sensory cortex (S1) and motor cortex (M1) in mice *in vivo* following single whisker stimulation. The results prove this mesoscopic imaging technique has the potential to serve as a useful tool in investigating the layer-specific neural connectivity in the mouse cortex *in vivo*.

## 2 Materials and Methods

All *in vivo* experiments were performed in accordance with the National Institutes of Health Guide for the Care and Use of Laboratory Animals (NIH Publication No. 80-23), approved by the Institutional Animal Care and Use Committee at the University of Maryland, Baltimore and College Park campuses.

### 2.1 Animal Preparation

Four male Thy1-ChR2-YFP mice (stock number: 007612, Jackson Laboratory) at 6 to 10 weeks of age were used. These Thy1-ChR2-YFP transgenic mice express the light-activated ion channel, channelrhodopsin-2 (from the green algae *Chlamydomonas reinhardtii*), fused to yellow fluorescent protein (ChR2-YFP) under the control of the mouse thymus cell antigen 1 (Thy1) promoter.^51^^–^^53^ The spatial distribution of ChR2 in the Thy1 mouse line has been well characterized in several previous studies.^51^^–^^53^ Within the brain, ChR2-positive cells appear in large pyramidal neurons in cortical layer 5, CA1 and CA3 pyramidal neurons in the hippocampus, mossy fibers in the cerebellum, and neurons in various regions of the thalamus, midbrain, and lower brainstem as previously reported.^51^^,^^53^ Within the cortex, ChR2 is mainly expressed in both axons and dendrites of the layer 5 pyramidal neurons that have pronounced apical dendritic tufts in layers 1 and 2/3.^18^^,^^51^^–^^54^ Also, it has been shown that ChR2 expression varies <50% across the anterior-posterior axis within layer 5.^55^ We noticed that there were some ChR2 expression in other cortical neurons as well. However, previous studies have validated that photostimulation was most effective in evoking action potentials in L5 pyramidal cells and less capable of evoking action potentials in pyramidal cells in other layers.^52^ Another four male B6 mice at 6 to 10 weeks of age were used to study the layer-specific functional projections between S1 and M1. Animals were anesthetized using urethane (1.15  g/kg body weight).^33^ The mouse head was shaved before securing in a stereotaxic frame (Stoelting Ltd.).^56^ The whiskers except C2 were trimmed in the right whisker pad of B6 mice. A cranial window [about 3 (medial–lateral) mm × 3 (anterior–posterior) mm] covering mainly S1 was made over the left parietal cortex for Thy1-ChR2-YFP mice. For B6 mice, the bone overlying the left parietal cortex was removed to expose both S1 and M1. Extreme care was taken during surgery to avoid damaging the cortex. Hemostatic sponge dipped in the artificial cerebrospinal fluid (ACSF) was applied to clean the surface of the dura matter. VSD RH-1691 (Optical Imaging Ltd.; 1.0  mg/ml in ACSF) was then applied to the exposed area for 90 min.^33^^,^^56^ After VSD staining, the cortex was washed with dye-free ACSF for 15 min to remove unbound dye. The cortical surface was subsequently covered with high-density silicone oil and sealed with a coverslip.^33^ The body temperature was maintained around 37±0.5°C using a heating blanket throughout the experiment. At the end of the experiment, mice were euthanized and transcardially perfused with 0.1 M phosphate buffer saline (pH 7.4) followed by 4% paraformaldehyde. Brain slices were examined to verify the expression of enhanced YFP-tagged ChR2 in the brains of the transgenic mice.

### 2.2 Imaging System

**Fig. 1:**
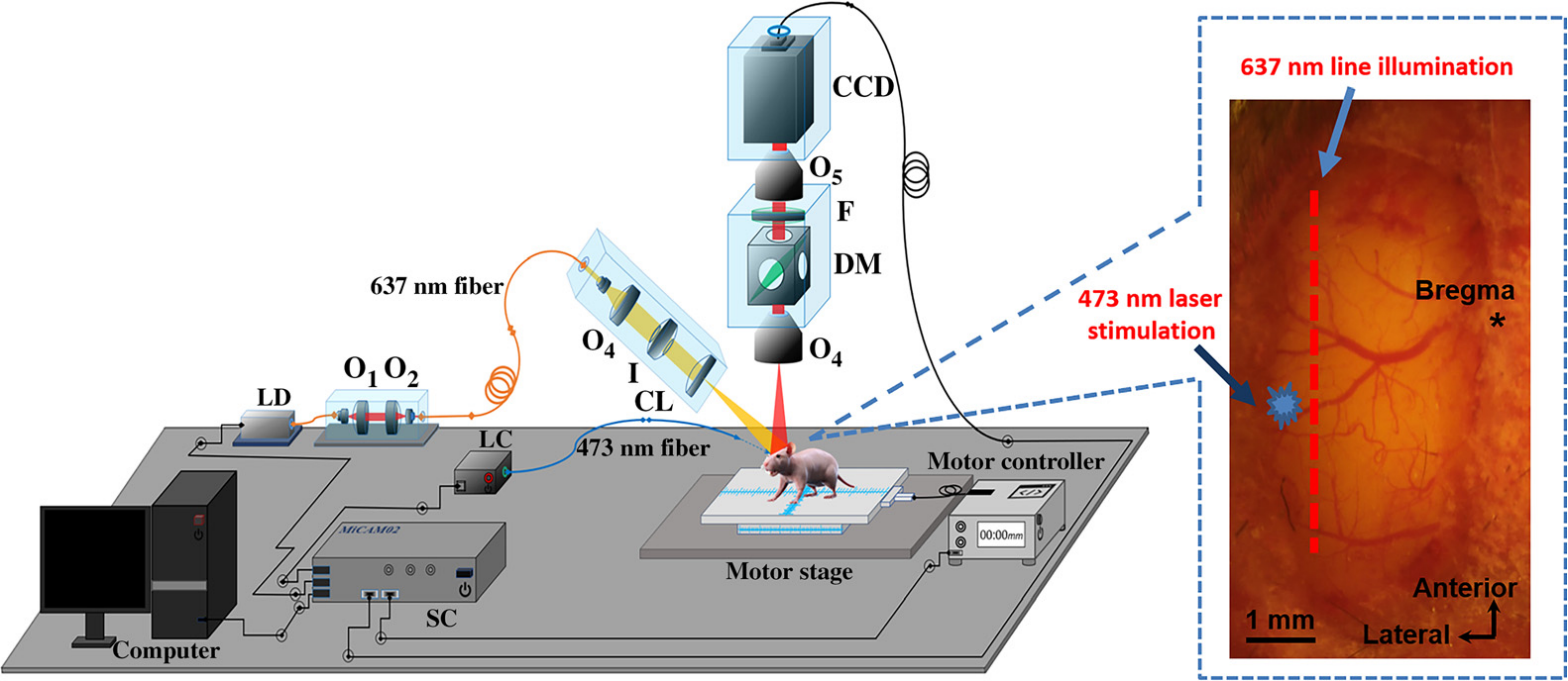
Schematic of the mesoscopic system. LD: laser diode; O: objective lens; I: iris; CL: cylindrical lens; F: filter; DM: dichroic mirror; LC: laser controller; and SC: synchronization controller. The illumination arm is arranged at 45 deg in air. The inset image shows the line illumination and the 473-nm laser stimulation on the mouse brain surface.

Figure 1 shows the schematic diagram of the mesoscopic imaging system. A 637-nm laser diode was used as the light source. The light was first collimated (O1) and coupled (O2) into a single-mode fiber to shape the light beam.^33^ Light coming out from the fiber was collimated by an objective lens (O3) and the collimated light was expanded into line-field illumination using a cylindrical lens (CL) with a full-line-width at the half-maximum of ∼26  μm at the focal plane. An iris was used to control the length of the line illumination.^33^ The emitted fluorescent light was collected back through an objective lens (O4), a dichroic mirror (650 nm, single-edge dichroic beam splitter; FF650-DiO1-50×70  mm; Andover Corporation), an emission filter (695 nm, 695FG07-50, Andover Corporation), another objective lens (O5), and finally imaged to a high-speed CCD camera (MiCAM02-HR, SciMedia Ltd.). The illumination angle was set at 45 deg, rendering ∼30-deg transmission angle in tissue (with n∼1.33).^33^ The CCD camera was vertically placed to record both fluorescence and reflectance images by changing the emission filter (no filter for reflectance recording and with emission filter for fluorescence imaging). A motor stage was used to translate the sample laterally in scanning direction (perpendicular to the line illumination direction).^57^ To photostimulate layer 5 pyramidal neurons in Thy1-ChR2-YFP mice, blue laser light (473 nm, MBL-FN-473 nm, Changchun New Industries Optoelectronics Tech. Co., Ltd.) was coupled into a single-mode fiber. The fiber was mounted on an XYZ manipulator and targeted at the cortical surface for photostimulation as shown in Fig. 1. To study the layer-specific functional projections between S1 and M1 in B6 mice, a glass pipe (1.0-mm in diameter) fitted onto an XYZ manipulator was aimed at the facial C2 whisker (not shown in Fig. 1). Air-puff stimulus was applied through a Picospritzer pressure valve connected to the glass pipette.^33^^,^^56^ The stimulation system (either the 473-nm laser or the air-puff stimulus system), the 637-nm illumination, the CCD camera, and the motor controller were synchronized through the synchronization controller box in the MiCAM02-HR imaging system (SciMedia, Ltd.).

### 2.3 Stimuli and Data Acquisition

To mainly photostimulate cortical neurons of S1 cortex in Thy1-ChR2-YFP mice, the 473-nm, blue laser light (fiber diameter: 200  μm, numerical aperture: 0.37) with intensity $∼50\;\mathrm{mW}/{\mathrm{mm}}^{2}$ and stimulus duration of 5 ms was used. The selection of the light intensity is based on the intensity decay of 473 nm light with a conical angle of 32 deg in mice brain.^58^ The minimum intensity required for generation of ChR2-evoked action potentials is greater than $∼1\;\mathrm{mW}/{\mathrm{mm}}^{2}$.^58^^–^^62^ With the current laser power and fiber-optic applied, this optical neural interface in principle can evoke spiking in neurons up to 0.93 mm from the fiber tip, which covers mainly the cortical tissues.^63^ The air-puff stimulus was set to be 20 ms duration for contralateral C2 whisker in B6 mice. In order to record 3D VSD dynamics, which reflect the cellular processes at the millisecond time scale,^48^ time-resolved acquisition protocol was implemented as previously described.^31^^,^^33^ The line-illumination light was first focused on the border of the desired FoV.^64^ At each scanning position, an experimental session including all the time related images was acquired. Specifically, at each illumination location, the experimental section consisted of 20 trials of recording. For the photostimulation experiment, each trial has 400 frames with 2.5  ms/frame. For the whisker stimulation to study the layer-specific functional projections between S1 and M1, each trial has 200 frames with 5  ms/frame. We set higher temporal resolution in the photostimulation experiment in order to image the dynamics of the layer interactions from L5 in the sensory cortex, while we maintained the high spatial resolution in the whisker experiment in order to image the response in the motor cortex (less camera pixels were used when taking images at 2.5  ms/frame). In each trial, the stimulus (473 nm blue laser light, 5 ms duration) or 20 ms whisker deflection was presented at time point of 500 ms (one stimulus per trial). Twenty trials of imaging were repeated to increase the SNR. The interval between each trial is 8 s for neural recovery. The images from these 20 trials were averaged to obtain the response $XYT_{i}S_{0}$ (where X=184; Y=128; i=1,…,200 for the whisker stimulation experiment; X=88; Y=60; i=1,…,400 for the photostimulation experiment) at the first scanning position $S_{0}$. During imaging, we set the focus plane slightly below the dural surface because of defocus effect. After this, the motor stage moved a step in the scanning direction and another experimental session was performed to obtain the dataset at this new illumination/collection area. This process was repeated until the entire FoV was covered. In the photostimulation experiment, we set 60 steps with a step size of 46  μm, which resulted in ∼2.7  mm total movement in scanning direction. In order to cover both the sensory and motor cortices, we set 60 steps with a step size of 66  μm resulting ∼3.96  mm total movement in scanning direction for the whisker experiment. Since the optical properties are different in air and brain, brain surface must be found to make sure the accuracy of the reconstruction.^33^ The last step for each experiment was to acquire reflectance images of the cortical surface. To obtain the surface tomography of the mice cortex, the emission filter was removed and the same FoV was imaged to get the reflectance images with the same scanning step size. Without emission filter, most of the collected signal came from the reflection of the illumination light at the brain surface, which would then serve as an indicator of the location of cortex surface.^33^ The raw measurement of reflectance mode had the format of $XYS_{j}$ (X=184; Y=128; j=1,…,60 for the whisker stimulation experiment; X=88; Y=60; j=1,…,60 for the photostimulation experiment).

### 2.4 Data Reconstruction and Analysis

The raw measurements of the reflectance data were simply stacked based on the geometrical relationship between the illumination plane and imaging plane,^33^ similar to the unprocessed stacked raw image in selective-plane illumination microscopy.^65^ The reconstruction process for 3D neural responses was similar to previously reported and the detailed mathematical expression for the reconstruction process can be found in our previous publications.^33^^,^^64^ Briefly, for each XYT dataset at each scanning position, the final 10 prestimulus frames (90 to 99th frame for whisker stimulation and 190 to 199th frame for photostimulation) were averaged as the baseline $F_{0}$. The baseline image was then subtracted from each subsequent frame $F_{t}$ to obtain changes in fluorescence signals ΔF, and relative changes in fluorescence [$\Delta F/F_{0}$ (%), ordinate] were used as our final measurements M.^33^ For the next step, the images at different scanning positions with the same time-frame number were rearranged as one data set (e.g., the dataset for first frame of the trial at all scanning positions was rearranged as $XYT_{1}S_{j}$ (where X=184; Y=128; j=1,…,60 for the whisker stimulation experiment; X=88; Y=60; j=1,…,60 for the photostimulation experiment).^33^ The CCD pixel size was ∼22  μm for the whisker experiment and ∼46  μm for the photostimulation experiment. For the whisker stimulation data sets, the step size in scanning direction needs to be matched to the CCD pixel size. Interpolation was done in the scanning dimension using a custom MATLAB algorithm changing the data sets to $XYT_{i}S_{j}$ (where X=184; Y=128; i=1,…,200; j=1,…,180). For the photostimulation experiment, since the CCD pixel size is the same as the step size, the data sets of ${XYT}_{i}S_{j}$ (where X=88; Y=60; i=1,…,400; j=1,…,60) remained unchanged. To reconstruct the images, we utilized the first-order Born approximation assuming a linear relationship between the measurement M and the fluorophore distribution C, which is the $\mathrm{FoV}\;x^{\prime }z^{\prime }$ to be reconstructed from the measured XS at each time point. This linear relationship can be written as M=JC, where J is the weight or sensitivity matrix.^33^ To constitute J, photon distribution was first generated by Monte-Carlo simulation (g=0.9, n=1.33, $\mu_{a}=0.01/\mathrm{mm}$, and $\mu_{s^{\prime }}=0.82/\mathrm{mm}$).^66^ The optical property of the mice brain was determined from the reflectance data using oblique-incidence spectroscopy.^33^^,^^67^ Next, we applied the reciprocity principle and J was later decomposed by singular value decomposition.^33^^,^^68^ Finally, least square fitting and Tikhonov regularization^37^ were applied to solve this underdetermined system.^33^^,^^37^ The regularization parameter of α=0.0016 was determined by L-curve criterion.^33^^,^^69^ For the photostimulation experiment, 60 source–detector pairs and 60 scanning positions were chosen to constitute 3600 measurements. Each reconstructed $\mathrm{FOV}\;x^{\prime }z^{\prime }$ consists of 60×60  pixels with a pixel size of ∼46  μm. Weight matrix J is therefore of size 3600×3600. For the whisker experiment, 160 source–detector pairs and 160 scanning positions were chosen to constitute 25,600 measurements. Each reconstructed FOV $x^{\prime }z^{\prime }$ consists of 160×160  pixels with a pixel size of ∼22  μm. Weight matrix J is therefore of size 25,600×25,600. FOV $x^{\prime }y^{\prime }z^{\prime }$ was constituted by superimposing individual $\mathrm{FOV}x^{\prime }z^{\prime }$ in Y direction. The ROI for fluorescence images to perform reconstruction were selected based on the cortex surface from the reflectance images using our custom MATLAB algorithm.^33^ This reconstruction process was performed at different time points to obtain the temporal 3D neural responses.

### 2.5 Statistical Analysis

Data are expressed as mean ± standard deviation. A two-sample t-test with unequal means was applied to determine whether the difference was significant in the statistical parameters between any two sample groups. Differences were regarded as statistically significant if p<0.05.

## 3 Results

### 3.1 Histological Characterization of the Chr2 Expression in Thy1-Chr2-YFP Mice

**Fig. 2:**
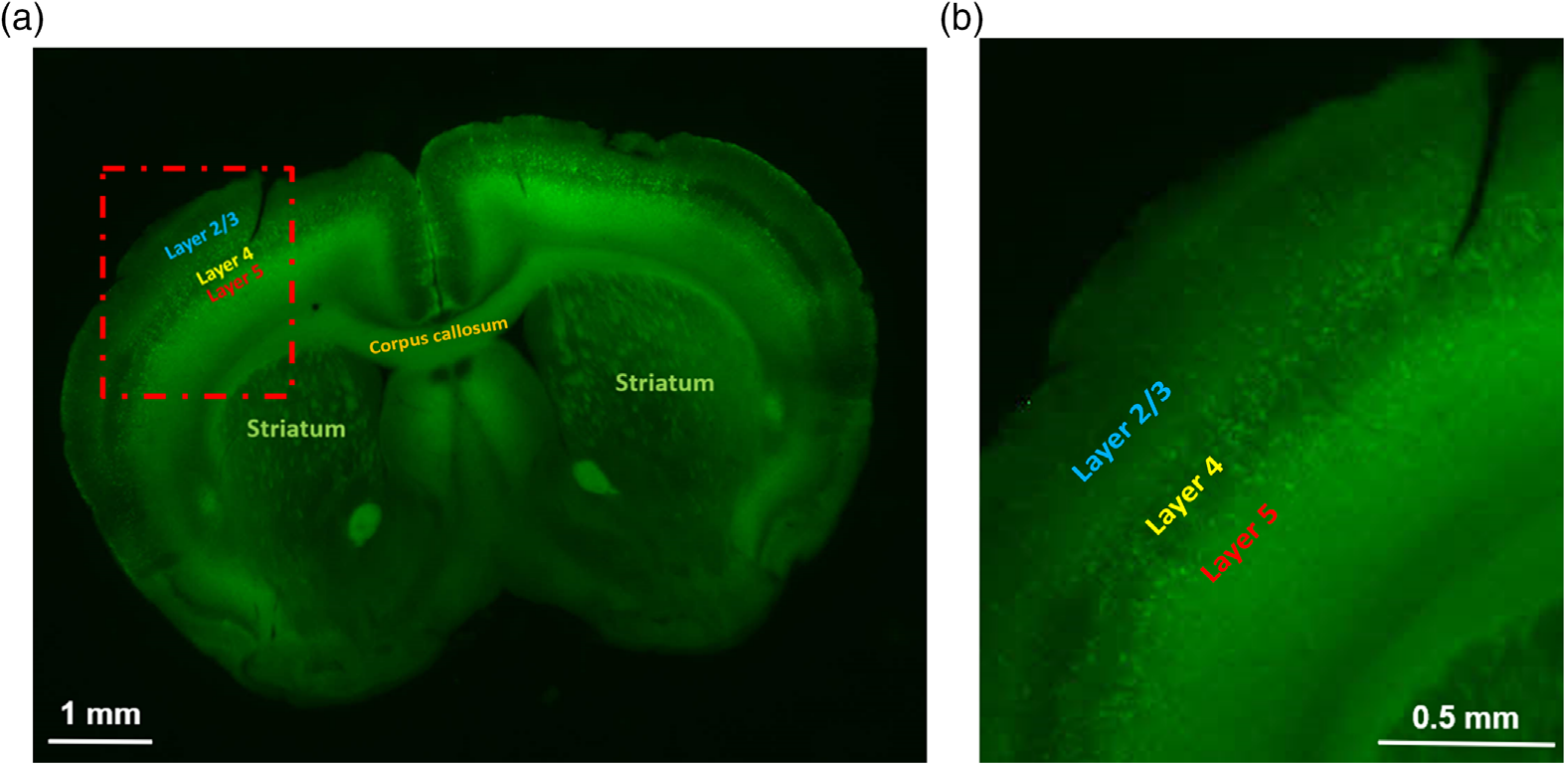
(a) Expression of channelrhodopsin-2 (ChR2) in Thy1-ChR2 mice. (b) Preferential expression of YFP-tagged ChR2 in layer 5 pyramidal neurons in the cortex. The image in the zoomed image is from the red dashed square in (a).

Histological characterization of the ChR2 expression by imaging YFP signal was performed in Thy1-Chr2-YFP mice. ChR2 expressed in many brain regions including cortical neurons, corpus callosum and in some deep structures of the brain as shown in Fig. 2(a). In the somatosensory cortex [Fig. 2(b)], ChR2 was expressed in pyramidal neurons and other cells in L5, with some expression in L2/3 and L4 neurons as well. However, previous studies have validated that photostimulation was most effective in evoking action potentials in L5 pyramidal cells and less capable of evoking action potentials in pyramidal cells in other layers.^52^

### 3.2 2D CCD Imaging after Photostimulation in the Somatosensory Cortex

**Fig. 3:**
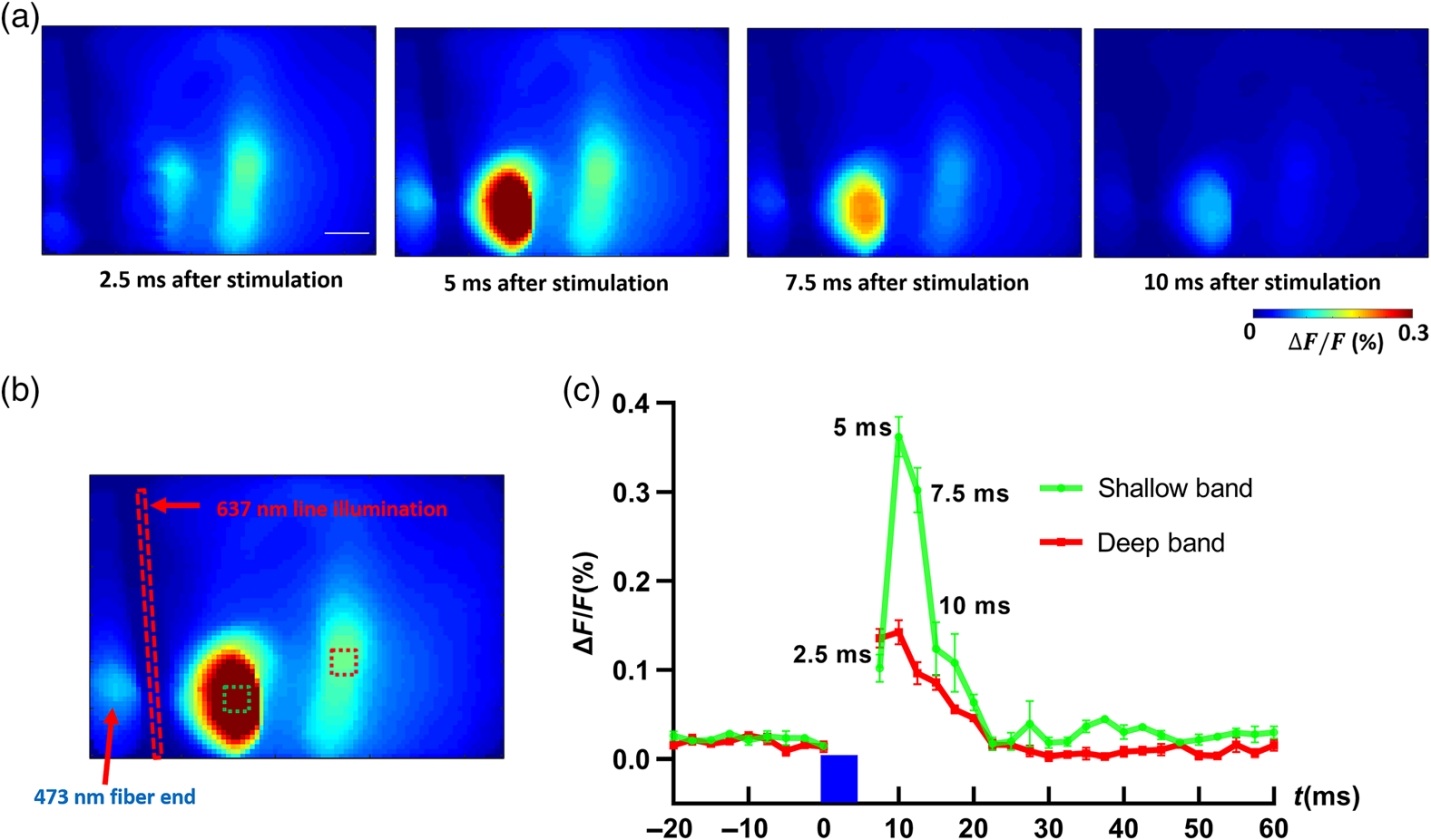
(a) 2D CCD measurements of changes in fluorescence [ΔF/F(%), ordinate] in response to the 5-ms 473 nm laser stimulation from oblique line illumination. Time period after stimulation is indicated at the bottom of each image. Scale bar: 0.5 mm. (b) Imaging schematic showing the location of the 473-nm laser stimulation and the 637-nm line illumination. (c) Changes in fluorescence [ΔF/F(%), ordinate] in response to the 5-ms 473 nm laser stimulation. Fluorescence signal was calculated from the ROIs (green square and red square: 5×5  pixels) shown in (b). The blue column indicates the 5-ms 473 nm laser stimulation. Time points corresponding to those shown in (a) are indicated near the green plot. N=4.

In the photostimulation experiment, 473 nm blue laser light was used to stimulate the cortical neurons in Thy1-ChR2-YFP mice. The brief flash (5 ms) allowed to minimize stimulation artifacts that could interfere with VSD imaging.^52^ The 473-nm blue laser stimulation could also excite the YFP and the VSD, because of this, the responses during the 5-ms blue light stimulation were discarded. Figure 3(b) indicates the location of the 473-nm laser stimulation, and the 637-nm line illumination. Since the line illumination was set 45 deg toward the right side of the image, most of the responses will appear at the right side of the line illumination. With this experimental setup, we used ChR2 to photostimulate cortical neurons and using VSD imaging to detect resulting responses throughout S1. Figure 3(a) shows the 2D CCD measurements of changes in fluorescence [ΔF/F(%), ordinate] at different time points after the photostimulation (at one representative scanning position). In general, the responses appeared as double bands shape with different distances to the line illumination location, corresponding to different depths in the cortex (the longer distance to the line illumination site represents deeper region in the cortex). The intensity of each band varied as time changed. At 2.5 ms after photostimulation, the deeper band (with longer distance from the illumination site) already appears large response area and strong intensity. After 2.5 ms (5 ms after photostimulation), the intensity and response area of the shallower band dramatically increased while the intensity and response area of the deeper band slightly increased. At 7.5 and 10 ms after photostimulation, the intensity and response area of both bands decreased. Figure 3(c) shows the quantitative change in fluorescence [ΔF/F(%)] in response to the 5-ms 473 nm laser stimulation at different time points. Fluorescence signal was calculated from the ROI (green square and red square: 5×5  pixels) shown in Fig. 3(b). Together with Fig. 3(a), the dynamics of response from both the deeper band and the shallower band can be clearly displayed.

### 3.3 Combining Photostimulation with 3D Mesoscopic Imaging in the Somatosensory Cortex

**Fig. 4:**
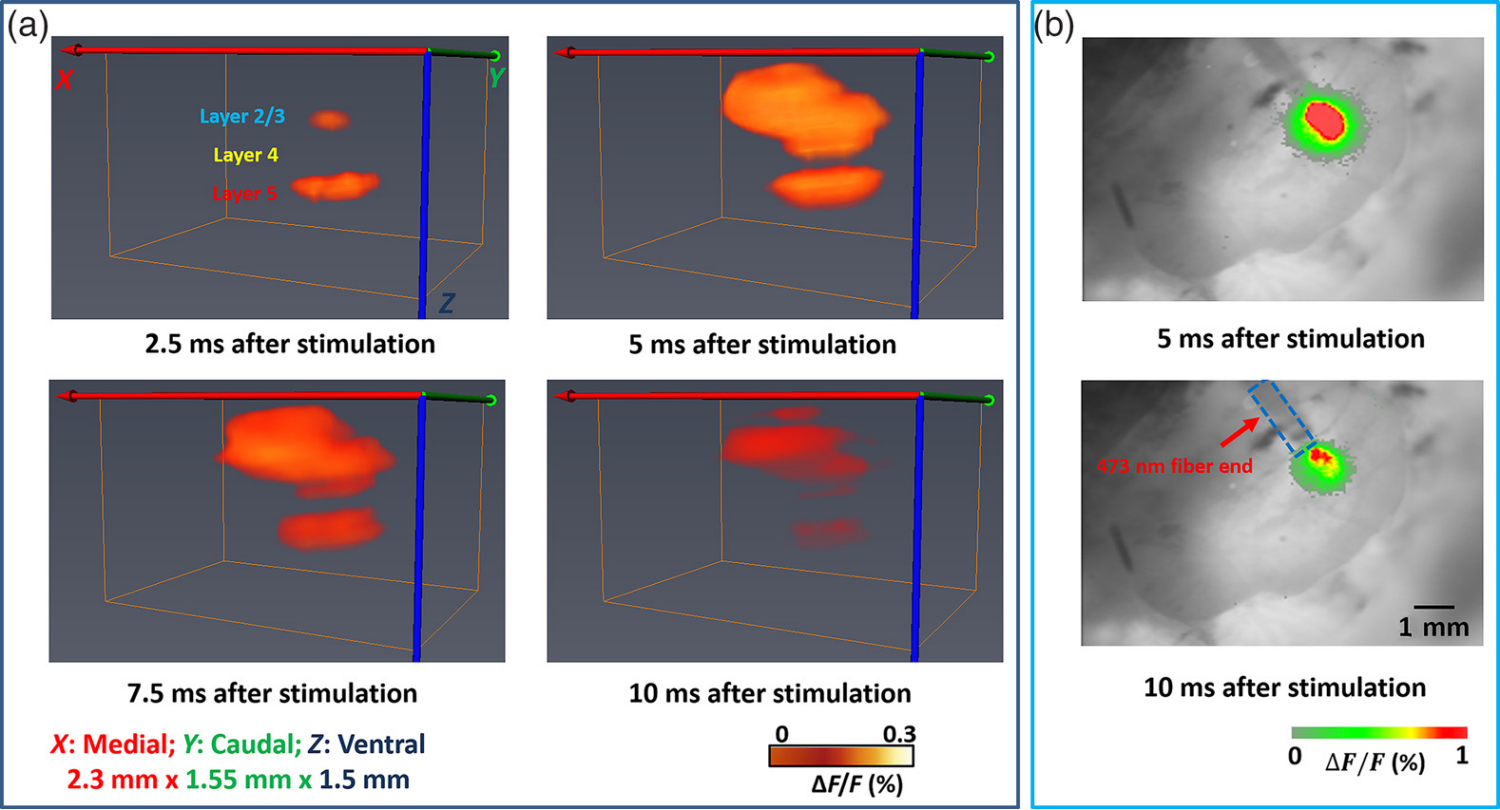
(a) 3D changes in fluorescence [ΔF/F(%), ordinate] in response to the 5-ms 473 nm laser stimulation reconstructed by the mesoscopic system. (b) Changes in fluorescence [ΔF/F(%), ordinate] in response to the 5-ms 473 nm laser stimulation captured by the conventional 2D planar imaging system. Time period after stimulation is indicated at the bottom of each image. Red arrow indicates the location of the 473-nm stimulation fiber.

After recording the data at all the scanning positions, 3D neural activities were reconstructed following the protocol in Sec. 2.4. As shown in Fig. 4(a), photostimulation initially evoked excitatory responses in deeper areas (corresponding to layer 5) [Fig. 4(a), top left] that peaked within 5 ms after photostimulation [Fig. 4(a), top right]. Excitatory responses subsequently spread up along the column into shallower areas (corresponding to layer 2/3) by 5 ms after photostimulation [Fig. 4(a), top right]. The excitatory responses maintained in both layer 5 and layer 2/3 until 10 ms after photostimulation [Fig. 4(a), bottom right]. Notably, the area between layer 2/3 and layer 5 (corresponding to layer 4) showed very weak signal, resulting to the overall “sandwich” structure along the cortical depth. In contrast, from the conventional 2D planar imaging, the activation pattern at 5 and 10 ms after photostimulation is shown in Fig. 4(b). The responses appeared surrounding the 473-nm stimulation fiber end, with decreased activation at 10 ms after stimulation. The time-course trend was similar to the 3D result, while since the signals from different depths were integrated, the 3D sandwich structure could not be resolved.

**Fig. 5:**
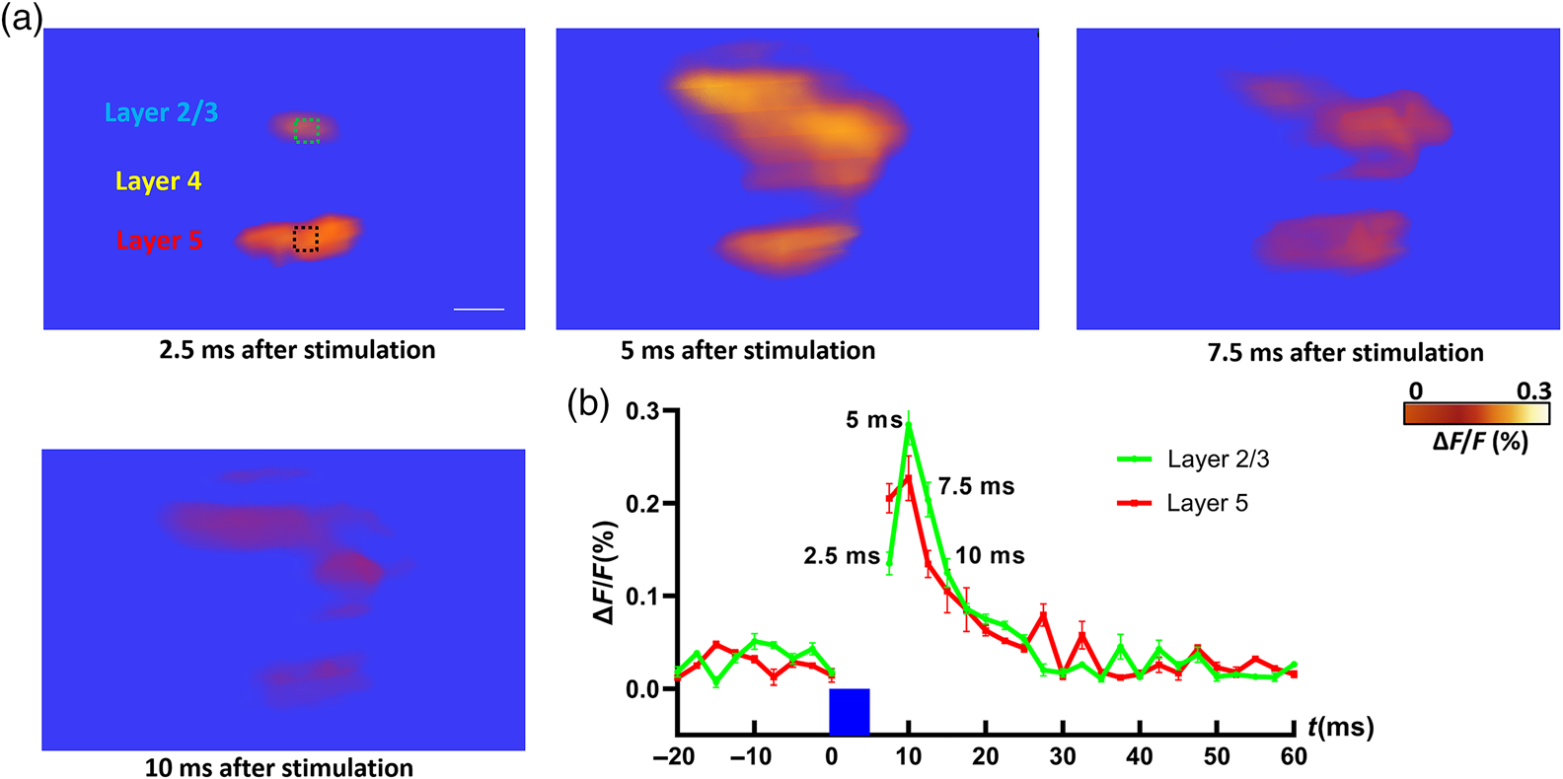
(a) 2D changes in fluorescence [ΔF/F(%), ordinate] in response to the 5-ms 473 nm laser stimulation reconstructed by the mesoscopic system in XZ cross section. Scale bar: 250  μm. (b) Changes in fluorescence [ΔF/F(%), ordinate] in response to the 5-ms 473 nm laser stimulation. Fluorescence signal was calculated from the ROIs (green square and black square: 5×5  pixels) shown in (a) at 2.5 ms after stimulation. The blue column indicates the 5-ms 473 nm laser stimulation. Time points corresponding to those shown in (a) are indicated near the green plot. N=4.

To better visualize the depth-resolved neural activities, 2D XZ cross sections of changes in fluorescence [ΔF/F(%), ordinate] were reconstructed as shown in Fig. 5(a). The neural response dynamics in different layers can be clearly resolved. XZ cross sections provide a new perspective to investigate the neural activities at different time points. Figure 5(b) further shows the quantitative change in fluorescence [ΔF/F(%)] in response to the 5-ms 473 nm laser stimulation at different time points for L2/3 and L5. Fluorescence signal was calculated from the ROI (green square and black square: 5×5  pixels) shown in Fig. 5(a). The results provide us the temporal and spatial dynamics of the neural responses after photostimulating cortical neurons in different regions of the sensory cortex. All the four animals imaged showed a similar trend.

### 3.4 3D Mesoscopic Imaging of Neural Connections in Sensory and Motor Cortices

**Fig. 6:**
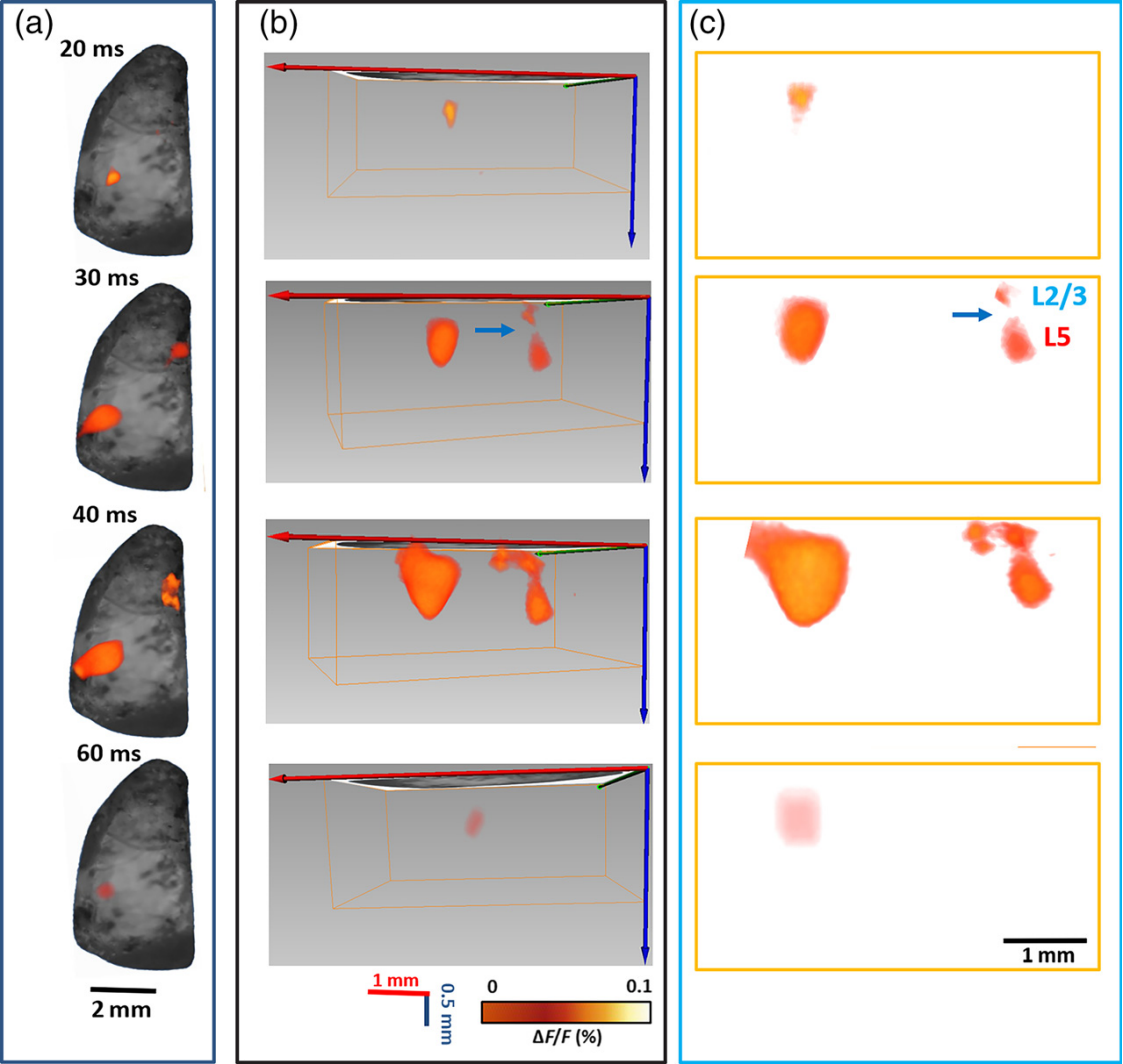
(a) 2D depth-integrated images of changes in fluorescence [ΔF/F(%), ordinate] in response to C2 whisker stimulation; (b) corresponding 3D fluorescence changes in brain; and (c) corresponding 2D fluorescence changes in XZ cross sections. Time period after stimulation is indicated at the left side of each image. Blue arrow indicates the distinct band between L5 and L2/3.

Next, we recorded the depth-resolved neural responses in sensory and motor cortices following single whisker stimulation. Figure 6 shows the images of neural responses evoked by contralateral C2 whisker stimulation. As shown in the 2D depth-integrated images in Fig. 6(a), a single-brief passive deflection of the C2 whisker evoked a stereotypical pattern of cortical activity imaged with VSD. The responses occurred first with a latency of 20 ms following whisker stimulation in sensory cortex and was highly localized, exciting the C2 barrel column of the S1 cortex specifically. Over the next 20 ms, the depolarization spread across a large part of the sensory barrel cortex, indicating that neurons from the surrounding barrel columns became depolarized. Approximately 10 ms after the earliest response in S1 cortex, a second localized anteromedial cortical region within the M1 cortex region was depolarized and spread over the following 10 ms. Finally, after ∼60  ms following whisker deflection, the evoked activity disappeared gradually. As previously reported,^70^ deflection of C2 whisker initiated cortical activities in two clearly separate focal regions, from which propagating waves of depolarization can spread to a large part of the sensorimotor cortex. However, imaging the voltage-dependent fluorescence changes of neocortex stained with VSD using a fast CCD can only reveal the 2D membrane potential dynamics. In contrast, using our mesoscopic imaging system, the 3D spatiotemporal dynamics of cortical activities can be reconstructed as shown in Fig. 6(b). The ability to resolve 3D spatiotemporal dynamics is of importance when considering the layer-specific functional connections between barrel cortex and motor cortex. At 30 ms in Fig. 6(b), we can notice the neural responses in M1 showed a sandwich structure with a distinct band between the two strong response regions. The two strong response regions were within the identified L2/3 and L5 location of the M1. The corresponding 2D fluorescence changes in XZ cross sections were further shown in Fig. 6(c). This 3D imaging method provided a new perspective about the layer-specific neural interactions between different functional areas within the cortex.

**Fig. 7:**
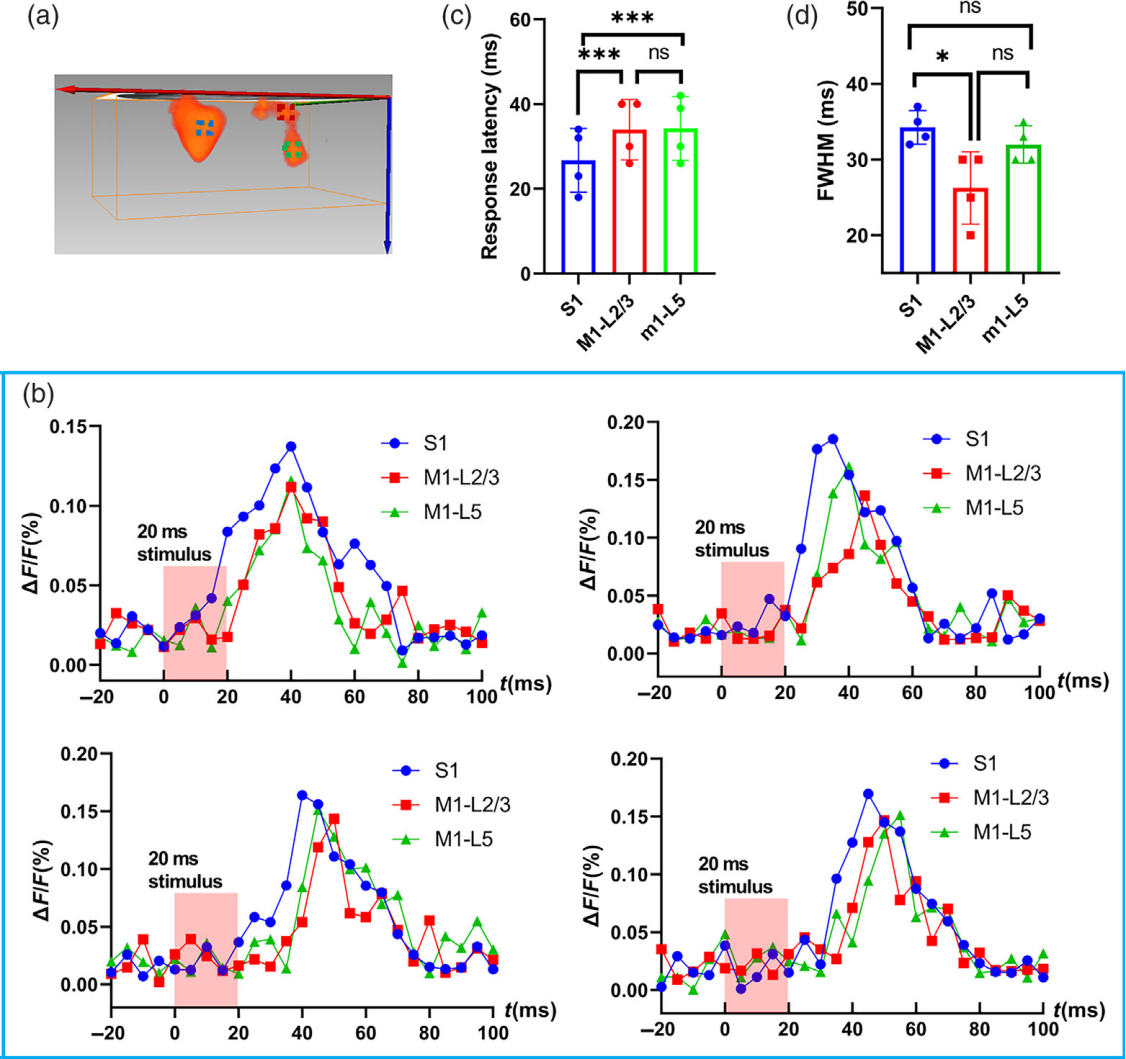
(a) Example image to showing the ROIs selected for calculation. (b) Changes in fluorescence [ΔF/F (%), ordinate] in response to the 20-ms whisker stimulation in four mice. Fluorescence signal was calculated from the ROIs (blue, red, and green cubic boxes: 5×5×5  pixels) shown in (a). The red column indicates the 20-ms whisker stimulation. (c) Comparison of the response latency in S1, L2/3 of M1, and L5 of M1. The response in S1 shows a significantly lower value than that in L2/3 and L5 of M1. (d) Comparison of the FWHM of the response in S1, L2/3 of M1, and L5 of M1. The response in S1 shows a significantly higher value than that in L2/3 of M1.

To study the response dynamics, we further plotted the change in fluorescence [ΔF/F(%)] in response to the 20-ms whisker stimulation at different time points for S1, L2/3 of M1, and L5 of M1 from all four mice as shown in Fig. 7(b). Fluorescence signal was calculated from the ROIs (blue, red, and green cubic boxes: 5×5×5  pixels) shown in Fig. 7(a). It is clear that the response in L2/3 of M1 and L5 of M1 appeared after that in S1. To quantitatively study the response latency in S1, L2/3 of M1, and L5 of M1, we defined the response latency as when the fluorescence signal reaches the half maximum value in the trial. The response latency in S1 shows a significantly lower value than that in L2/3 and L5 of M1, while there is no significant difference in response latency between L2/3 of M1 and L5 of M1. The latency is 7.5±0.5  ms (n=4) between the response in S1 and M1. We further defined the full-width at half-maximum (FWHM) of the response as the duration of the activation. The FWHM in S1 shows a significantly higher value than that in L2/3 of M1, while there is no significant difference in FWHM between S1 and L5 of M1, and between L2/3 of M1 and L5 of M1.

## 4 Discussion

Visualization of evoked and spontaneous neuronal activity *in vivo* is of importance for understanding brain functions. Sensory pathways in the mammalian brain have been particular targets of imaging studies, because neural networks can be evaluated following specific stimulation of the sense organs, which translate and transmit physical energy to the brain. The neocortex serves as the central part of processing and responding to specific sensory input.^71^ Cortical layering is a hallmark of the mammalian neocortex and a major determinant of local synaptic circuit organization in neural systems^13^. Due to the limited thickness of mouse cortex (with 6 layers in ∼1  mm^63^), it raises a big challenge to study the layer-specific microcircuits in the cortex. Conventional optical methods for functional brain imaging based on planar CCD could provide widefield imaging of the aggregate neural activity^72^ or mesoscopic imaging at single-cell resolution using sophisticated genetic tools^73^ or structured illumination schemes,^74^ while does not have depth-resolved capability. At the microscopic scale, confocal and two-photon microscopy can provide cellular spatial resolution, while have limited imaging depth, FoV, and temporal resolution especially for 3D functional imaging. Although we need to notice, there are emerging improvements for multi-photon microscopy in imaging depth,^75^^,^^76^ large FoV,^77^^–^^80^ and imaging speed.^81^^–^^83^ At the macroscopic scale, fMRI and DOT have been applied to achieve 3D reconstruction of cerebral hemodynamics in small animals.^23^^–^^25^^,^^84^^–^^87^ The macroscopic imaging methods can provide a large FoV and imaging depth (some can cover the whole mouse brain) while the spatial resolution is limited. Moreover, the relationship between BOLD signals and underlying brain activities remains unclear.^88^ Photoacoustic tomography (PAT), which starts with optical absorption by tissue molecules and ends with ultrasonic emission through thermoelastic expansion, has been demonstrated in brain functional imaging from microscopic to macroscopic scales.^89^^–^^91^ However, PAT is also an indirect measure of neural activity in the brain, focusing on assessment of hemodynamics and oxygen metabolism in mouse cortical vasculatures. To image neural responses at different cortical layers in mouse brain cortex, an imaging method in the mesoscopic range, which can visualize the layer-specific interactions that are too deep for the microscopy to image and too small for the macroscopy to resolve is needed. LOT is developed to image absorption contrast for hemodynamic changes in mesoscopic range and has been demonstrated to record the vascular compartment dynamics during somatosensory stimulation.^29^^,^^92^ However, due to the sparse sources/detectors configuration and the absorption contrast, the previously reported mesoscopic methods still have limited spatial resolution and cannot provide the direct neural responses measurements. In this paper, we demonstrated an improved mesoscopic 3D imaging method using an oblique illumination/detection system configuration and high-density sources/detectors (line illumination and CCD camera). Line illumination could speed up the image acquisition compared to point scanning. VSD was combined to this 3D imaging method to visualize the direct layer-specific responses after specific stimulus in mouse cortex.

We first tested the depth-resolved capability of our imaging method using well-characterized Thy1-ChR2-YFP mice. Using transgenic mice and photostimulation, it is possible to stimulate specific cortical neurons. From the raw CCD recording in Fig. 3, we can see that with oblique illumination/detection configuration alone, neural responses at different depths can be resolved. Since the raw measurements do not take photon distribution and detector sensitivity into consideration, the signal quantification is not accurate enough. For instance, there will be more excitation photons in the shallower regions such as L2/3 compared to deep regions such as L5. Through image reconstruction, 3D neural responses in the cortex can be obtained. The nature of reconstruction can help to improve the signal quantification accuracy at different depths by taking photon distribution and sensitivity of the detectors into consideration.^93^ At 2.5 ms after photostimulation, both the raw recordings (Fig. 3) and reconstructed results (Figs. 4 and 5) indicate that the deeper region (L5) already appears large response area and strong intensity.

It is not surprising to see this since within the cortex, ChR2 is mainly expressed in the L5 pyramidal neurons and the photostimulation could preferentially activated the L5 pyramidal neurons.^18^^,^^51^^–^^54^ We can see there was also responses from L2/3 at 2.5 ms after photostimulation. One possibility for the responses may be the sparse ChR2-labeled neurons in L2/3 and the apical dendrites of L5 cells as shown in Fig. 2, though the previous study found that photostimulation was most effective in evoking action potentials in L5 pyramidal cells and less capable of evoking action potentials in pyramidal cells in other layers.^52^ Another possibility is that the signal of L2/3 at 2.5 ms after photostimulation was from the excitation of L5 neuron since the excitation of L5 neurons started at 0 ms and we did not have the recording until 7.5 ms after photostimulation started. At 5 ms after photostimulation, the intensity and response area of the deeper region (L5) slightly increased. On the other hand, the intensity and response area of the shallower band dramatically increased at 5 ms. At 7.5 and 10 ms after photostimulation, the intensity and response area of both regions decreased. Since ΔF/F was used to display the neural activation in this study, ΔF/F is a good parameter to compare dynamics, but its amplitude is not an appropriate index when comparing fluorescence from different depths considering uneven VSD staining. Although one interesting observation about the signal dynamics is that the intensities of L2/3 and L5 were flipped at 5 ms after photostimulation and lasted until the excitation ended. The consistent observed signal inversion (observed in all four mice) could indicate the dynamics of signal transmission. We suspect that the photostimulation preferentially activated the pyramidal neurons in L5, L5 pyramidal neurons excited L2/3 pyramidal neurons, as well as other L5 pyramidal neurons in neighboring columns, which is consistent with the known connectivity pathways of L5 pyramidal neurons.^52^^,^^94^ Since the pyramidal neurons in L5 directly innervate L2/3 pyramidal neurons, there was very weak signal in L4, which resulted into a sandwich structure. In contrast, the conventional VSDi method can only provide a depth-integrated image since it does not have the depth-resolved capability as shown in Fig. 4(b). Another observation is that that the light evoked duration of activation pattern (Fig. 4, ∼10  ms) lasted shorter than that induced by whisker (Fig. 6, ∼40  ms), which agrees well that previously reported.^95^ The combination of optogenetics with the 3D mesoscopic imaging method could provide a new possibility to study the 3D functional neuronal connections after stimulating/inhibiting neurons of certain locations or certain types. In order to study the layer-specific functional circuitry, we will combine cell type-specific optogenetics with our mesoscopic imaging techniques for next step.

We then imaged the 3D functional neural connectivity between sensory and motor cortices in the rodent whisker-barrel system, one sensory pathway that is highly amenable to experimental manipulations.^2^^,^^33^ Rodents use their whiskers to locate and identify objects.^96^ Forces acting on whiskers excite sensory neurons in the trigeminal ganglion, triggering activity that ascends through the brainstem into ventral posteromedial thalamic nucleus and L4 neurons in the primary somatosensory barrel cortex (S1).^4^^,^^5^ L4 stellate cells mainly excite layers L2/3 pyramidal neurons, which in turn excite neurons in L5.^4^^,^^5^ A subset of L2/3 and L5 neurons in S1 project to primary motor cortex (M1, M1 also has layered structure similar to S1, with different types of neurons in each layer). The S1 and M1 are reciprocally connected, and their interaction has long been hypothesized to contribute to coordinated motor output. However, very little is known about the nature and synaptic properties of the S1 input to M1.^97^ Neuroanatomical tracing experiments have often been used to predict circuits and have shown that L2/3 pyramidal neurons in S1 had the densest innervation of deeper layers 5/6 in M1, whereas the L5 pyramidal neurons in S1 preferentially innervated the superficial layers of M1.^4^^,^^5^ However, axodendritic overlap is not necessarily a good predictor of functional connection strength.^4^^,^^47^ It will be of great interest to examine the functional consequences of the layer-specific projections from S1 to M1, which can help reveal the primary loci where sensorimotor associations are formed and will provide the possibility to serve as a tool with which one can learn more about brain disease processes and the effects of treatment.^4^ Since our mesoscopic imaging method uses line illumination to scan the FoV, we can easily adjust the length of the line beam to cover both the sensory and motor cortices simultaneously. The 3D spatiotemporal dynamics of cortical activities in both S1 and M1 can be imaged (Fig. 6). The sandwich structure with a distinct band between L5 and L2/3 in M1 can help to reveal the 3D neuronal distribution in M1 receiving inputs from S1, which is promising considering it is within intact cortex in living mice. The distinct band between L5 and L2/3 has also been demonstrated in the *ex vivo* brain slice studies,^4^^,^^97^ and a recent study has indicated that the neurons located in a thin laminar zone at the L3/5A border form the genuine layer 4 in motor cortex.^98^ The ability to resolve 3D spatiotemporal dynamics is of importance when considering the functional connections between barrel cortex and vibrissal motor cortex since it can provide us a clue about the strength of S1 input as a function of cortical layer. The 7.5±0.5  ms (n=4) latency difference between S1 and M1 activity is consistent with the recordings from the 2D imaging^70^ and consistent with a pyramidal neuron axonal conduction velocity of ∼450  μs/ms.^99^ For ∼3.5  mm separation of S1 and M1, the time required for action potential propagation is ∼7.78  ms. The duration of the activation in S1 shows a significantly higher value than that in L2/3 of M1, but not L5 of M1. The early response in S1 and feedback loop linking S1 and M1 may explain the longer duration of activation in S1 compared to L2/3 of M1.^4^ Since the L5 neurons in M1 will further interact with the motor centers in the brainstem after sensorimotor integration,^100^ it may cause the duration of the activation time vary. We need to note that there are only four mice in this study, which is not enough to come to a solid result. More mice should be included to get a statistical conclusion. In this study, the response in motor cortex is induced by deflecting the C2 whisker on the contralateral snout. Thus it is conveyed by inputs from both L2/3 and L5 in S1.^5^ In order to determine the separate contributions of L2/3 and L5 neurons to activating targets in M1, it is necessary to excite or suppress certain layer in S1 and observe the 3D functional responses in M1. In the future, we will combine the optogenetic control strategy with the 3D mesoscopic imaging method to further determine the separate contributions of L2/3 and L5 neurons to activating targets in M1.

Several potential improvements of this mesoscopic imaging technique can be identified. First, the distribution of VSD is not homogenous across different depths in the cortex, although we allowed sufficient time for the VSD to diffuse. A possible alternative solution would be using genetically encoded voltage indicators,^101^^–^^110^ which will make it possible for cell class specific targeting thus enabling non-invasive longitudinal studies. Surface flatness is another factor that limits the reconstruction accuracy especially when imaging a large FoV, since we assumed the surface being flat in our reconstruction model. The exact shape of the brain surface needs to be extracted in future studies. The assumption of homogeneous optical properties in the reconstruction could potentially limit the accuracy of reconstruction results although the absorption and scattering properties of different types of biological tissues are relatively homogeneous.^111^ An iterative procedure that uses determined optical properties to calculate new fits of signal versus effective source–detector separation might allow for better empirical optimization of both optical properties and further improve the accuracy of the reconstruction model.^25^ Moreover, photon migration estimation using the mathematical models could not be exact, especially for complicated biological tissues.^64^^,^^112^^,^^113^ The path of photons become more difficult to predict as they scatter further. The mesoscopic methods face resolution deterioration of these reconstructed images as a function of depth.^30^^,^^33^^,^^112^ The problem can be alleviated by combining dense spatial data sets with regularization terms like compressive sensing-based methods.^114^ The quantitative accuracy and penetration depth can also be improved by incorporating the high-dynamic-range method reported previously.^115^^,^^116^ In addition, the synthesis of improved long-wavelength VSD has the potential to further enhance the imaging penetration depth.^117^^–^^119^

In this paper, the time-resolved acquisition protocol has been applied to record the fast neural dynamics, while it requires the biological response to be repeatable for each stimulation trial, which potentially limits the scope of potential applications. We should note that the long-time repetitions could possibly induce long-term plasticity in the neural network. There are two reasons for applying the time-resolved acquisition protocol. First, compared to hemodynamics imaging based on absorption contrast (slow signal changes at second time scale), VSDi reports the neural activity in the brain with millisecond time scale.^48^ Second, the amplitude of change in fluorescence for the response is relatively low (<0.5%) using VSD, which makes it necessary to perform tens of averaging for one recording. Genetically encoded voltage indicators mentioned above could be a possible solution assuming that the SNR can be significantly increased compared with the currently used VSD, which can help reducing the averaging times. In the future, we will also explore other fluorescent dyes that can indicate the neural responses by contrast such as changes in ion concentrations (pH-, calcium-, chloride-, or potassium-sensitive dyes).^48^^,^^120^^–^^123^ These fluorescent dyes indicating changes in ion concentrations happen in seconds time scale and can provide better SNR, which has the potential to significantly reduce the acquisition time for our mesoscopic system and improve the reconstruction accuracy.

In summary, we document that our mesoscopic imaging method could map layer-specific functional regions and their connections. In combination with optogenetic control, the ability to image 3D neuronal responses in the mouse neocortex with high temporal and spatial resolution will yield a wealth of information about circuit organization and function. This methodology has the potential to examine the functional consequences of disrupted functional connectivity in diseased animal models (e.g., genetic and surgical disruptions).
